# Persistence of IgG COVID-19 antibodies: A longitudinal analysis

**DOI:** 10.3389/fpubh.2022.1069898

**Published:** 2023-01-10

**Authors:** Álvaro Carvalho, Ana Rita Henriques, Paula Queirós, Joana Rodrigues, Nuno Mendonça, Ana Maria Rodrigues, Helena Canhão, Germano de Sousa, Francisco Antunes, Miguel Guimarães

**Affiliations:** ^1^Fundação Álvaro Carvalho, Lisboa, Portugal; ^2^CHRC, NOVA Medical School, Universidade NOVA de Lisboa, Lisboa, Portugal; ^3^Fundação Vox Populi, Lisboa, Portugal; ^4^Germano de Sousa Group- Centro de Medicina Laboratorial, Pólo Tecnológico de Lisboa, Lisboa, Portugal; ^5^Instituto de Saúde Ambiental, Faculdade de Medicina, Universidade de Lisboa, Lisboa, Portugal; ^6^Laboratório Associado TERRA, Faculdade de Medicina, Universidade de Lisboa, Lisboa, Portugal; ^7^Ordem dos Médicos Portugueses, Lisboa, Portugal

**Keywords:** COVID-19, SARS-CoV-2, antibody responses, IgG, humoral immune response, post-infection immunity

## Abstract

**Background and aim:**

The kinetics of antibody production in response to coronavirus disease 2019 (COVID-19) infection is not well-defined yet. This study aimed to evaluate the antibody responses to SARS-CoV-2 and its dynamics during 9-months in a cohort of patients infected during the first phase of the pandemic. As a secondary aim, it was intended to evaluate the factors associated with different concentrations of IgG antibodies.

**Methods:**

A prospective cohort study was conducted from June 2020 to January 2021. This study recruited a convenience sample of adult individuals who where recently diagnosed with COVID-19 and were living in mainland Portugal. A total of 1,695 blood samples were collected from 585 recovered COVID-19 patients up to 9 months after SARS-CoV-2 acute infection. A blood sample was collected at baseline and three, 6 and 9 months after SARS-CoV-2 acute infection to assess the concentration of IgG antibody against SARS-CoV-2.

**Results:**

The positivity rate of IgG reached 77.7% in the first 3 months after symptom onset. The IgG persists at all subsequent follow-up time-points, which was 87.7 and 89.2% in the 6th and 9th months after symptom onset, respectively. Three distinct kinetics of antibody response were found within the 9 months after infection. Kinetic 1 (K1) was characterized by a constant low IgG antibody concentration kinetic (group size: 65.2%); kinetic 2 (K2), composed by constant moderate IgG kinetic (group size: 27.5%) and kinetic 3 (K3) characterized by higher IgG kinetic (group size: 7.3%). People with ≥56 years old (OR: 3.33; CI 95%: [1.64; 6.67]; *p*-value: 0.001) and symptomatic COVID-19 (OR: 2.08; CI 95%: [1.08; 4.00]; *p*-value: 0.031) had higher odds of a “Moderate IgG kinetic.” No significant association were found regarding the “Higher IgG kinetic.”

**Conclusion:**

Our results demonstrate a lasting anti-spike (anti-S) IgG antibody response at least 9 months after infection in the majority of patients with COVID-19. Younger participants with asymptomatic disease have lower IgG antibody positivity and possibly more susceptible to reinfection. This information contributes to expanding knowledge of SARS-CoV-2 immune response and has direct implications in the adoption of preventive strategies and public health policies.

## Introduction

In late 2019, the World Health Organization (WHO) was officially informed of the occurrence of a pneumonia cluster in the city of Wuhan, China (1). It was a new Coronavirus – Severe Acute Respiratory Syndrome Coronavirus 2 or SARS-CoV-2 (2). Considering the rapid spread to several countries globally, in March 2020, the World Health Organization declared the SARS-CoV-2 virus a global pandemic (3). However, the disease came to be called Coronavirus Diseases 2019, commonly known as COVID-19 (2).

As of September 25, 2022, a total of 615 million confirmed cases and 6,54 million deaths had been reported worldwide (4). COVID-19 shows a complex profile with many different clinical features. Clinical manifestations can ranging from asymptomatic infection to severe inflammatory syndrome and multiorgan dysfunction. The majority of symptomatic infections result in mild-COVID-19, with fever, sore throat, cough, myalgia, and/or malaise, and with lower frequency gastrointestinal symptoms and loss of taste or smell, but without shortness of breath, and dyspnea (5–9). Patients with severe disease (oxygen saturation < 94%, or respiratory rate of >30 breath/min, or lung infiltrates >50%) or critical disease (respiratory failure, septic shock and/or multiorgan failure) accounted for up to 14% of cases and in about 5% of cases, respectively (10). Male sex, age >55 years, multiple pre-existing comorbidities and obesity (body mass index (BMI) >30 kg/m^2^) appear commonly associated with increased disease severity and/or mortality (6).

In humans, three classes of antibodies or immunoglobulins have been the target of SARS-CoV-2 serological tests: IgM, IgG and IgA (11). Among the three classes of antibodies, our study focused on IgG. This antibody is often the most abundant in the serum and plasma, and have higher specificities when compared with assays that detect IgM and IgG and plays a more prominent role after first 2–3 weeks following acute infection, and establishing long-term immune memory, that can persist for several months or years (12, 13).

It is essential to know the human immune response against SARS-CoV-2. However, the kinetics of antibody production in response to COVID-19 infection is not well-defined yet. Knowledge gaps exist regarding the persistence of immune response after infections and kinetics of immune antibody production according to disease severity. Although the limited number of published data from experimental and clinical research, the results suggest that post-infection humoral immunity may protect against SARS-CoV-2 reinfection (14–17). The durability of the humoral immune response is not well-defined yet. To date, the most prolonged observation period assessing the longevity of the antibody response has been 12 months (18). However, so far, most studies are being assessed up to 6–8 months after disease onset (19–22).

This study aimed to evaluate the IgG antibody responses to SARS-CoV-2 and its dynamics during 9-months in a cohort of patients infected during the first phase of the pandemic (03–05/2020). For this purpose, samples were collected at three, 6 and 9 months after COVID-19 infection. As a secondary aim, it was intended to evaluate the factors associated with severe disease and different concentrations of antibodies.

## Methodology

### Study design and recruitment

A prospective cohort study was conducted from June 2020 to January 2021. This study recruited a convenience sample of adult individuals who had been recently diagnosed (between 1 to 7 months before) with COVID-19 and were living in mainland Portugal. Patients were contacted to participate in the study in person, by phone or by email. At baseline, participants who signed an informed consent were then asked to answer a self-administered paper and pencil questionnaire and to collect a blood sample to measure IgG antibody concentration against SARS-CoV-2. During follow-up a blood sample was collected at three, 6 and 9 months after acute SARS-CoV-2 infection.

### Study population

Adults (18 years old), Portuguese speakers, residing in Portugal mainland, infected with SARS-CoV-2. Participants vaccinated against COVID-19 during the study were excluded.

### Measurement, assessment, and instruments

#### Baseline self-administered questionnaire

The baseline questionnaire was composed of questions regarding sociodemographic (age, sex, level of education, area of residence, household size, professional situation, and workplace); anthropometric data (self-reported weight and height), BMI calculated as kg/m^2^ and categorized into two levels (Underweight/Normal – BMI < 24.9 kg/m^2^; Overweight/Obese – BMI ≥25 kg/m^2^); smoking habits (daily; in the past; never); presence of chronic non-communicable diseases (“Do you suffer from any of the following chronic diseases?” - diabetes, arterial hypertension, other cardiovascular diseases, respiratory disease, chronic kidney disease, oncological disease, autoimmune disease, other); COVID-19 symptoms (“Have you had one of these symptoms?” – fever, cough, muscle pain, headache, shortness of breath, loss of taste or loss of smell); place of isolation during COVID-19 (at home; at hospital), if yes to isolation in hospitalized- “How many days?,” “In which unit?” – nursery, ICU unventilated, ICU ventilated; epidemiological history: contact with a person infected with SARS-CoV-2 (Yes; No) if yes in contact with a person infected with SARS-CoV-2, inside or outside the household, - influenza vaccine (Yes; No). A trained person from the research team was always present to clarify possible doubts that the participants might have when answering the questionnaire.

### Laboratory procedures

A blood sample was collected at baseline and three, 6 and 9 months after COVID-19 to assess the concentration of IgG antibody against SARS-CoV-2. A 5 mL blood sample was collected from each participant. Serological laboratory analysis was performed for all samples. Venous blood was collected in BD Vacutainer^®^ SST^®^ tubes, which contain a clot activator and separator gel to obtain serum. The tests used were the LIAISON^®^ SARS-CoV-2 IgG tests from DiaSorin, performed on Liaison XL. All tests met the requirements of the manual of good laboratory practices and were carried out in duplicate whenever required to confirm the result. A chemiluminescence immunoassay was used to test participants for antibodies to SARS-CoV-2. The test detects and quantifies anti-SARS-CoV-2-IgG antibodies produced in response to the spike (anti-S) protein, produced in the context of infection. A case was considered seropositive for SARS-CoV-2 when concentrations of anti-SARS-CoV-2 IgG antibodies were ≥ to 15 U/mL, as established by the manufacturer (DiaSorin) (23). All reagents used were *in vitro* diagnostic products that were subject to previous performance tests. Internal validation of the reagents was performed for all tests (correlation tests). The test has a clinical sensitivity of 98.7% and a clinical specificity of 99.5%.

### Outcomes definition and measurments

**Figure 1:**
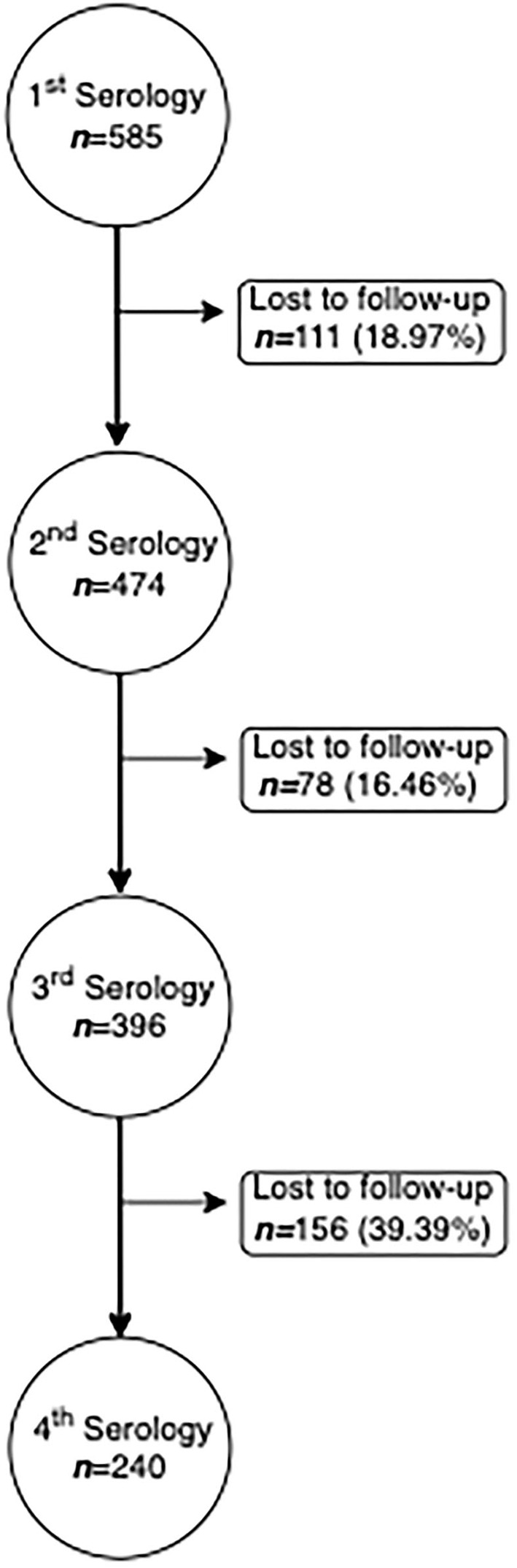
Flowchart of the study.

The primary outcome of this study was the concentration of IgG antibodies against SARS-CoV-2.Three follow-up time-points were considered: 3 months (0–3.99), 6 months (4–6.99), and 9 months (7–9.99) after symptoms onset (Figure 1). Baseline was defined as the date of the first blood collection.

For the second aim, the outcome was disease severity, divided into three groups according to the characteristics of the disease: (a) Asymptomatic, mild to moderate – patients who did not require hospitalization; (b) severe – patients who were hospitalized; (c) critical – patients who were hospitalized in an intensive care unit (ICU).

### Covariates of interest

Sociodemographic (age, sex, level of education, area of residence, household size, professional situation and workplace); anthropometric data (self-reported weight and height), BMI calculated as kg/m^2^ and categorized into two levels (Underweight/Normal – BMI < 24.9 kg/m^2^; Overweight/Obese – BMI ≥25 kg/m^2^); smoking habits (daily; in the past; never); presence of chronic non-communicable diseases (diabetes, arterial hypertension, other cardiovascular diseases, respiratory disease, chronic kidney disease, oncological disease, autoimmune disease, other); COVID-19 symptoms (fever, cough, muscle pain, headache, shortness of breath, loss of taste or loss of smell); place of isolation during COVID-19 (at home; at hospital), days of hospitalization, hospital unit (nursery, ICU unventilated, ICU ventilated); epidemiological history: contact with a person infected with SARS-CoV-2 (Yes; No) contact with a person infected with SARS-CoV-2 (inside or outside the household), influenza vaccine (Yes; No).

### Statistical analysis

Descriptive data for each categorical variable was presented as the absolute frequency and the correspondent proportion. For continuous variables, the mean and standard deviation was presented. The patients were stratified by “with antibodies” and “without antibodies,” and the groups were compared by Student's *t*-test, chi-square and Fisher's exact tests. Participants were also stratified by disease severity (asymptomatic, mild to moderate, severe, and critical), and the groups were compared by Kruskal-Wallis test and chi-square test.

To assess the risk factors associated with disease severity, an ordinal logistic regression model was used. The response variable was the severity of the disease (asymptomatic, mild to moderate, severe, and critical), having as dependent variables the factors most commonly appointed as risk factors for severe disease – sex, age group (18–35; 36–55, ≥56), BMI (underweight or normal, overweight or obese) and the number of self-reported comorbidities (0–1, ≥2).

Group-based trajectory models (GBTMs) were used to derive the optimum number of antibody kinetics from zero to 9 months after symptom onset. Maximum likelihood was used to estimate the model parameters and mean disability count, following a censored normal distribution. The optimum number of disabilities and model fit was assessed using the Bayesian Information Criteria. Further, the logged bayes factor (2 ΔBIC) provided the strength of evidence against the simpler model (model with lesser groups).

The association between the factors commonly associated with a higher IgG antibody level (sex, age group, BMI, number of comorbidities, type of disease and severity) at baseline, and with antibody concentration kinetics (K1–K3) were examined using multinomial logistic regression. For this analysis, we only consider participants with two or more samples.

The IgG antibody concentration in each time-point was presented as median (interquartile range), the positive rate was presented as the absolute frequency and the corresponding proportion. The results of IgG antibody level and positive rate were presented stratified by sex (men, women), age group (18–35; 36–55, ≥56), and severity (asymptomatic, mild to moderate, severe, and critical).

Box-plots of the levels of IgG antibody and the plot displaying the IgG antibodies kinetics were created with the use of R software. All other analyses were performed using Stata IC, version 16.1 (StataCorp LP, College Station, TX, USA). Statistical significance was established as *p* < 0.05.

### Ethical issues

The study was carried out following the Declaration of Helsinki principles (24). Prior to the study beginning, an approval from the National Ethical and Deontological Committee of the Portuguese Medical Association (Conselho Nacional de Ética e Deontologia da Ordem dos Médicos) was received. All participants who agreed to take part in the study gave their written informed consent prior to their participation.

## Results

### Participants' baseline characteristics

A total of 585 participants with recent SARS-CoV-2 infection were enrolled in this study. All participants had already recovered from COVID-19 at baseline. The characteristics of the participants are described in Tables 1–3. Briefly, 445 (76.1%) were women and 140 (23.9%) men, with an average age of 47.9 years (18–96 years). The most participant's household (67.7%) was composed of three or more elements. The predominant levels of education were high school and higher education (74.2%); most participants were working full time (84.4%) and continue to work in their usual place (94.3%) during the pandemic. Participants were from different Portuguese regions, however, most of them were from the North (36.4%) and Lisbon (32.9%). Regarding lifestyles, most participants never smoked (72.3%). About self-reported chronic non-communicable diseases, the most frequent were hypertension (14.4%) and respiratory diseases (7.5%).

**Table 1:** Characteristics of the 585 participants at baseline, by IgG concentration.

	**Total *n =* 585**	**Antibody concentrations < 15 *n =*139**	**Antibody concentrations ≥15 *n =*446**	***p*-value**
**Sex**				0.11^a^
Women	445 (76.1%)	113 (81.3%)	332 (74.4%)	
Men	140 (23.9%)	26 (18.7%)	114 (25.6%)	
**Age, y (mean** **±sd)**	47.9± 15.5	45.3± 13.5	48.7± 16.0	**0.03** ^**b**^
**Age group (y)**				0.017^c^
18–35	132 (22.6%)	32 (23.0%)	100 (22.4%)	
36–55	286 (48.9%)	80 (57.6%)	206 (46.2%)	
56–96	167 (28.6%)	27 (19.4%)	140 (31.4%)	
**Household composition**				0.218^c^
1	51 (9.6%)	9 (6.9%)	42 (10.5%)	
2	120 (22.6%)	27 (20.8%)	93 (23.3%)	
3	164 (31.1%)	37 (28.5%)	128 (32.0%)	
≥4	194 (36.6%)	57 (43.9%)	137 (34.3%)	
**Education level**				0.036^c^
0–9 years	151 (25.8%)	28 (20.1%)	123 (27.6%)	
10–12 years	136 (23.3%)	27 (19.4%)	109 (24.4%)	
>12 years	298 (50.9%)	84 (60.4%)	214 (48.0%)	
**Employment status**				0.076^c^
Full time active worker	494 (84.4%)	124 (89.2%)	370 (83.0%)	
Other	91 (15.6%)	15 (10.8%)	76 (17.0%)	
**Workplace during pandemic** ^d^				0.363^c^
Workplace	461 (94.3%)	113 (91.9%)	348 (95.1%)	
Teleworking	18 (3.7%)	7 (5.7%)	11 (3.0%)	
Others	10 (2.1%)	3 (2.4%)	7 (1.9%)	
**Region**				0.171^c^
Norte	209 (36.4%)	52 (33.0%)	157 (35.9%)	
Centro	133 (23.2%)	24 (17.5%)	109 (24.9%)	
Lisboa	189 (32.9%)	53 (38.7%)	136 (31.1%)	
Alentejo/Algarve	43 (7.5%)	8 (5.8%)	35 (8.0%)	
**BMI categories**				0.234^c^
Underweight/Normal weight (BMI < 24.99 kg/m^2^)	288 (49.6%)	74 (54.0%)	214 (48.2%)	
Overweight/Obese (BMI ≥25 kg/m^2^**)**	293 (50.4%)	63 (46.0%)	230 (51.8%)	
**Smoking habits**				**0.001** ^**c**^
Smoker	51 (8.8%)	23 (17.0%)	28 (6.3%)	
Smoker in the past	109 (18.9%)	21 (15.6%)	88 (19.9%)	
Non-smoker	417 (72.3%)	91 (67.4%)	326 (73.8%)	
Yes	211 (36.3%)	49 (35.3%)	162 (37.0%)	
No	366 (63.4%)	90 (64.8%)	276 (63.0%)	
**Contact COVID-19**				**< 0.001** ^**c**^
Within the household	242 (41.6%)	39 (28.3%)	203 (45.7%)	
Outside the household	317 (54.5%)	90 (65.2%)	227 (51.1%)	
Without contact	23 (4.0%)	9 (6.5%)	14 (3.2%)	

^a^Fisher's exact test; ^b^*t*-test; ^c^Pearson chi-square; ^d^Workplace during the pandemic only for active workers.

Statistically significant values are denoted in bold.

Sample size is not constant due to missing data; Household composition refers to the number of people living in a single household. BMI, body mass index.

**Total:** Sex (*n* = 585); Age (*n* = 585); Age group (*n* = 585); Household composition (*n* = 530); Education level (*n* = 585); Place of abode (*n* = 574); Employment status (*n* = 585); Workplace during the pandemic (*n* = 489); BMI Cat (*n* = 581); Smoking habits (*n* = 577); Influenza vaccine (*n* = 577); Contact COVID-19 (*n* = 582).

**Antibody concentrations** **< 15**- Sex (*n* = 139); Age (*n* = 139); Age group (*n* = 139); Household composition (*n* = 130); Education level (*n* = 139); Place of abode (*n* = 137); Employment status (*n* = 139); Workplace during the pandemic (*n* = 123); BMI Cat (*n* = 137); Smoking habits (*n* = 135); Influenza vaccine (*n* = 139); Contact COVID-19 (*n* = 138).

**Antibody concentrations** **≥15** - Sex (*n* = 446); Age (*n* = 446); Age group (*n* = 446); Household composition (*n* = 400); Education level (*n* = 446); Place of abode (*n* = 437); Employment status (*n* = 446); Workplace during the pandemic (*n* = 366); BMI Cat (*n* = 444); Smoking habits (*n* = 442); Influenza vaccine (*n* = 438); Contact COVID-19 (*n* = 444).

**Table 2:** Chronic self-reported diseases of the 584 participants at baseline, by antibody concentration.

	**Total *n =* 585**	**Antibody concentrations < 15 *n =* 139**	**Antibody concentrations ≥15 *n =* 446**	***p*-value**
**Multimorbidity**				0.410^a^
0–1	463 (89.2%)	114 (91.2%)	349 (88.6%)	
≥2	56 (10.8%)	11 (8.8%)	45 (11.4%)	
Diabetes (yes)	28 (4.8%)	5 (3.6%)	23 (5.2%)	0.649^a^
High blood pressure (yes)	84 (14.4%)	10 (7.2%)	75 (16.8%)	**0.004** ^a^
Cardiovascular (yes)	25 (4.3%)	2 (1.4%)	22 (4.9%)	0.089^a^
Respiratory diseases (yes)	44 (7.5%)	14 (10.1%)	30 (6.7%)	0.199^a^
Chronic renal failure (yes)	9 (1.5%)	1 (0.7%)	8 (1.8%)	0.693^a^
Cancer (yes)	11 (1.9%)	1 (0.72%)	10 (2.2%)	0.473^a^
Autoimmune Diseases (yes)	29 (5.0%)	8 (5.8%)	21 (4.7%)	0.655^a^
Others (yes)	80 (13.7%)	16 (11.5%)	64 (14.4%)	0.48^a^

^a^Fisher's exact test.

Statistically significant values are denoted in bold.

Sample size is not constant due to missing data.

**Total:** Number of comorbidities (*n* = 519); Diabetes (*n* = 519); High blood pressure (*n* = 519); Cardiovascular (*n* = 519); Respiratory diseases (*n* = 519); Chronic renal failure (*n* = 519); Cancer (*n* = 519); Autoimmune Diseases (*n* = 519); Others (*n* = 519).

**Antibody concentrations** **< 15**: Number of comorbidities (*n* = 125); Diabetes (*n* = 125); High blood pressure (*n* = 125); Cardiovascular (*n* = 125); Respiratory diseases (*n* = 125); Chronic renal failure (*n* = 125); Cancer (*n* = 125); Autoimmune Diseases (*n* = 125); Obesity (*n* = 125); Others (*n* = 125).

**Antibody concentrations** **≥15**: Number of comorbidities (*n* = 394); Diabetes (*n* = 394); High blood pressure (*n* = 394); Cardiovascular (*n* = 394); Respiratory diseases (*n* = 394); Chronic renal failure (*n* = 394); Cancer (*n* = 394); Autoimmune Diseases (*n* = 394); Others (*n* = 394).

**Table 3:** Specific characteristics related to COVID-19, by antibody concentration.

	**Total *n=*585**	**Antibody concentrations < 15 *n =* 139**	**Antibody concentrations ≥15 *n =* 446**	***p*-value**
**Disease onset date**				
March 2020	382 (65.4%)	86 (61.9%)	296 (66.5%)	
April 2020	150 (25.7%)	38 (27.3%)	112 (25.2%)	
May 2020	26 (4.5%)	8 (5.8%)	18 (4.0%)	
June 2020	26 (4.5%)	7 (5.0%)	19 (4.3%)	
**COVID-19 symptoms**				**0.033** ^**b**^
Asymptomatic	109 (18.7%)	35 (25.2%)	74 (16.6%)	
Symptomatic	475 (81.3%)	104 (74.8%)	371 (83.4%)	
**Number of symptoms**				**< 0.001** ^**c**^
0–2	290 (49.6%)	91 (65.5%)	199 (44.6%)	
3–4	201 (34.4%)	35 (25.2%)	166 (37.2%)	
5–6	94 (16.1%)	13 (9.4%)	81 (18.2%)	
Fever ^e^	228 (48.0%)	31 (29.8%)	197 (53.0%)	**< 0.001** ^a^
Cough ^e^	265 (55.8%)	52 (50.0%)	213 (57.3%)	0.219^a^
Muscle pain ^e^	312 (65.6%)	58 (55.8%)	254 (68.3%)	**0.02** ^a^
Headache ^e^	272 (57.1%)	49 (47.1%)	223 (60.0%)	**0.025** ^a^
Shortness of breath ^e^	92 (19.3%)	19 (18.3%)	73 (19.6%)	0.888^a^
Loss of taste or smell ^e^	310 (65.1%)	58 (55.8%)	252 (67.7%)	**0.027** ^a^
**COVID-19 severity**				0.094^c^
Asymptomatic, Mild to Moderate	534 (91.8%)	132 (95.7%)	402 (90.5%)	
Severe	38 (6.5%)	6 (4.4%)	32 (7.2%)	
Critical	10 (1.7%)	-	10 (2.3%)	
**Isolation/treatment site**				0.1^a^
House	534 (91.8%)	132 (95.7%)	402 (90.5%)	
Hospitalized	48 (8.3%)	6 (4.4%)	42 (9.5%)	
**Number of days in hospital** ^d^ (mean ± sd)	14.3 ± 12.5	8.2 ± 6.4	15.1 ± 12.9	**0.006** ^b^
**Comparing to your health before**				**0.019** ^**c**^
**getting sick with COVID-19, how**				
**do you feel now**				
Better	53 (9.1%)	11 (8.0%)	42 (9.5%)	
Equal	377 (65.0%)	103 (74.6%)	274 (62.0%)	
Worst	150 (25.9%)	24 (17.4%)	126 (28.5%)	

^a^Fisher's exact test; ^b^*t*-test; ^c^Pearson chi-square, ^d^Fever, Cough, Muscle pain, Headache, Shortness of breath and loss of taste or loss of smell only for participants with symptomatic disease; ^e^Number of days in hospital, only for participants who were hospitalized.

Statistically significant values are denoted in bold. Entries are *n* (%) unless otherwise stated.

Sample size is not constant due to missing data.

**Total:** Disease onset date (*n* = 584); Type of disease (*n* = 585); Fever (*n* = 475); Cough (*n* = 475); Muscle pain (*n* = 475); Headache (*n* = 475); Shortness of breath (*n* = 475); Loss of taste or loss of smell (n=475); Severity (*n* = 578); Isolation/treatment site (*n* = 582); Number of days in hospital (*n* = 48); Health condition (*n* = 580).

**Antibody concentrations** **< 15:** Disease onset date (*n* = 139); Type of disease (*n* = 139); Fever (*n* = 104); Cough (*n* = 104); Muscle pain (*n* = 104); Headache (*n* = 104); Shortness of breath (*n* = 104); Loss of taste or loss of smell (*n* = 104); Severity (*n* = 138); Isolation/treatment site (*n* = 138); Number of days in hospital (*n* = 6); Health condition (*n* = 138).

**Antibody concentrations** **≥15:** Disease onset date (*n* = 445); Type of disease (*n* = 445); Fever (*n* = 371); Cough (*n* = 371); Muscle pain (*n* = 371); Headache (*n* = 104); Shortness of breath (*n* = 371); Loss of taste or loss of smell (*n* = 371); Severity (*n* = 440); Isolation/treatment site (*n* = 444); Number of days in hospital (*n* = 42); Health condition (*n* = 442).

Most participants were infected during the first phase of the pandemic between March and April 2020 (91%). In most cases, the COVID-19 was symptomatic (81.3%). The most reported symptoms were muscle pain (65.6%), followed by loss of smell and taste (65.2%), headache (57.1%), cough (55.7%) and fever (48.0%).

Regarding disease severity, the majority of participants ranged from asymptomatic, mild to moderate disease (91.8%), followed by severe (6.5%) and finally critical disease present in only 10 participants (1.7%). Most participants (91.8%) were isolated/recovered from the infection at home. However, 48 (8.3%) participants were hospitalized, 38 in the nursery and 10 in the ICU. Participants were on average hospitalized 14.3 ± 12.5 days.

When we look for factors associated with severe illness using ordered logistic regression (Supplementary Table 1), a statistically significant positive association was found between severity and sex (OR = 3.38; CI 95%= 1.76;6.51; *p*-value < 0.001), BMI (OR=2.13; CI 95% = 1.01;4.51; *p*-value = 0.049) and multimorbidity (OR = 2.78; CI 95% = 1.23;6.28; *p*-value = 0.014).

### The positivity rates of IgG anti SARS-CoV-2 antibody at three, sixth and nine months of follow-up

A total of 1,685 serial blood samples of 585 COVID-19 patients were tested for anti-IgG SARS-CoV-2 specific antibodies. The prevalence of IgG antibodies at each time-point is shown in Table 4. The positivity rate of IgG reached 77.7% in the first 3 months after symptom onset. One hundred forty-five patients did not show IgG antibody seroconversion at the first time-point. Among these 145 patients, there was no cases of critical disease, 136 (94.4%) ranged asymptomatic, mild to moderate disease, and 8 (5.6%) have a severe disease; about symptoms, there was the two types of patients in this group, 42.0 (29.0%) asymptomatic and 103 (71.1%) symptomatic; women were predominant in this group of patients (84.1%), as is the age group 36–55 years (56.6%) and BMI underweight or normal (54.9%).

**Table 4:** Temporal changes in the positive rate for IgG antibodies.

		**Positive rate *n* (%)**
**3 months (** ***n** **=*** **6 50)**		505 (77.7%)
	**Sex**	
	Women (*n* = 504)	382 (75.8%)
	Men (*n* = 146)	123 (84.3%)
	**Age group (y)**	
	18–35 (*n* = 148)	115 (77.7%)
	36–55 (*n* = 322)	240 (74.5%)
	≥56 (*n* = 180)	150 (83.3%)
	**Severity**	
	Asymptomatic, mild to moderate (*n* = 607)	471 (77.6%)
	Severe (*n* = 34)	26 (76.5%)
	Critical (*n* = 6)	6 (100%)
**6 months (*****n** **=*** **648)**		568 (87.7%)
	**Sex**	
	Women (*n* = 504)	440 (87.3%)
	Men (*n* = 144)	128 (88.9%)
	**Age group (y)**	
	18–35 (*n* = 130)	133 (86.9%)
	36–55 (*n* = 318)	276 (86.8%)
	≥56 (*n* = 200)	179 (89.5%)
	**Severity**	
	Asymptomatic, mild to moderate (*n* = 585)	512 (87.5%)
	Severe (*n* = 48)	43 (89.6%)
	Critical (*n* = 9)	9 (100%)
**9 months (*****n** **=*** **387)**		345 (89.2%)
	**Sex**	
	Women (*n* = 294)	259 (88.1%)
	Men (*n* = 93)	86 (92.5%)
	**Age group (y)**	
	18–35 (*n* = 70)	60 (85.7%)
	36–55 (*n* = 195)	174 (89.2%)
	≥56 (*n* = 122)	111 (91.0%)
	**Severity**	
	Asymptomatic, mild to moderate (*n* = 352)	314 (89.2%)
	Severe (*n* = 27)	23 (85.2%)
	Critical (*n* = 6)	6 (100%)

Anti-SARS-CoV-2 IgG antibody remained present at all subsequent follow-up time-points, which was 87.7 and 89.2% in the 6th and 9th months after symptom onset, respectively.

The positivity rate at each time point was presented, considering the variables: sex, age group, and severity (Table 4). Regarding sex, the biggest differences in the positivity rate are visible at the first time-points (75.8% in women vs. 84.3% in men). In the remaining time-points considered, the values were similar for men and women, close to 90%. For the age groups, the positivity rate was lower at the first time-point in the three groups considered (18–35 years; 36–55 years and ≥56 years), from 6 to 9 months, the positivity rate is similar in the three groups, although slightly lower in younger ones, in the last period there was a decrease in participants aged 18–35 years. Finally, to disease severity, all participants with critical disease had a concentration >15 (positive rate = 100%) in the three time-points considered. The positivity rate for severe and mild to moderate disease, is similar in both groups, at all time-points (Table 4).

### IgG antibody levels at three, sixth and nine months of following-up

**Figure 2:**
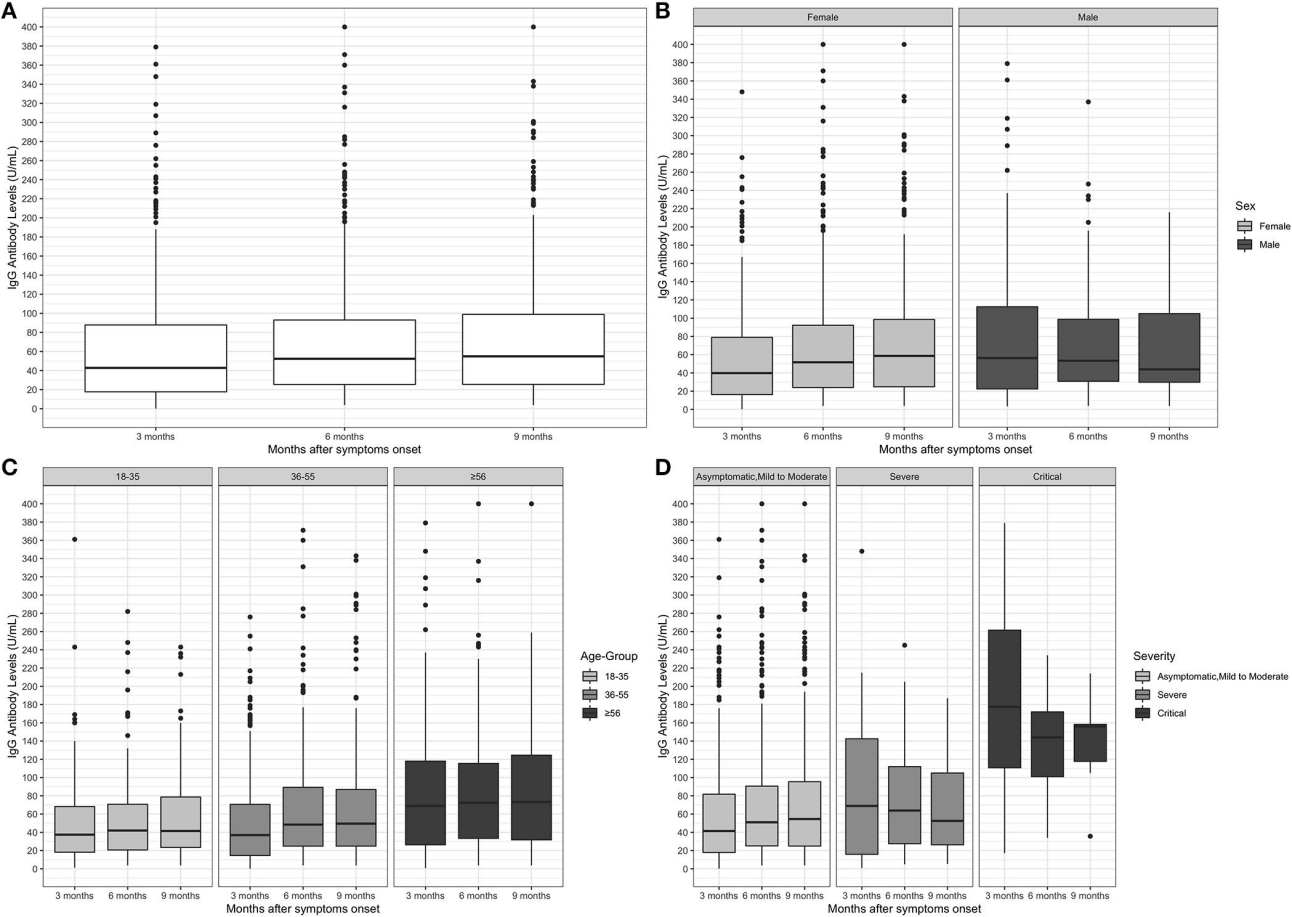
Antibody levels at each considered time-point. **(A)** total, **(B)** stratified by gender, **(C)** stratified by age-group; **(D)** stratified by disease severity. **(A)** The median IgG antibody level was 42.8 (IQR, 17.7–87.8) 3 months after symptom onset, 52.4 (IQR, 25.4–93.0) and 54.9 (IQR, 25.4–98.9) in the 6th and 9th months after symptom onset. **(B)** At the first time-point (three months), the IgG antibody levels were significantly higher in men compared to women (*p* = 0.003); for the remaining time-points, six (*p* = 0.776) and nine (*p* = 0.232) months, no significant differences were found. **(C)** The IgG antibody levels were significantly higher in the age group 56 years in the three time periods considered, compared to the age groups 18–35 (*p* ≤ 0.001; *p* ≤ 0.001; *p* = 0.005) and 36–55 (*p* ≤ 0.001; *p* ≤ 0.001; *p* = 0.005). **(D)** The IgG antibody levels were significantly higher in the critical severity level in the three time periods considered, compared to the asymptomatic, mild to moderate (*p* = 0.003; *p* ≤ 0.001; *p* = 0.005) and severe levels (*p* = 0.020; *p* = 0.008; *p* = 0.014).

We tracked the kinetics changes in IgG antibody levels for up to 9 months after symptom onset. The trend of antibody level changes was presented in Figure 2A. The median IgG antibody level was 42.8 (IQR, 17.7–87.8) 3 months after symptom onset, and the levels of IgG antibody was stable, which was 52.4 (IQR, 25.4–93.0) and 54.9 (IQR, 25.4–98.9) in the 6th and 9 months after symptom onset.

As for the positivity rate, the antibody kinetics was evaluated by: sex, age group, and severity (Figures 2B–D). In men, the median IgG antibody level was 56.3 (IQR, 22.4–113.0) at the first time-point, 53.4 (IQR, 30.8–99.5) at the second and 43.9 (IQR, 29.8–105.0) at the third. In women, the median IgG antibody level was 39.8 (IQR, 16.2-79.1) at the first time-point, 51.7 (IQR, 24.0–92.3) at the second and 58.7 (IQR, 24.8–99.3) at the third. At the first time-point, the median of IgG antibody level was lower in women than in men, coming closer at the other time-points. In the age group 18–35 years at the first time-point, the median IgG antibody level was 37.4 (IQR, 18-68.5), 42.0 (IQR, 20.7–70.8), and 41.5 (IQR, 23.3–80.3) at the second and third time-points respectively, in participants aged 36–55 years, the median was 36.95 (IQR, 14.6–70.7) in the first time-point, 48.5 (IQR, 24.8–89.5) in the second and 49.5 (IQR, 24.8–87.2) in the third, in the older participants (aged 56 or more years) the median IgG antibody level was 69.0 (IQR, 26.1–118.0), 72.4 (IQR, 33.3–116.0) at the second and 73.3 (IQR, 31.2–125.0) in the third time-point. It is noticeable that the antibody level was higher in older age groups than in younger ones. Finally, the levels of IgG antibodies seems to be proportional to the severity of the disease- the greater the severity, the greater the levels of antibodies. The median IgG antibody levels was 41.4 (IQR, 17.8–82.4) at the first time-point, 51.0 (IQR, 25.1–90.6) at the second and 54.6 (IQR, 24.9–95.7) at the third in asymptomatic, mild to moderate disease; in severe disease, the median IgG antibody is 68.9 (IQR, 15.7-146.0) at the first time-point, 63.9 (IQR, 26.7–112.0) at the second and 52.5 (IQR, 25.7–106.0) at the third; in critical disease, the median IgG antibody level was 177.5 (IQR, 89.0–289.0) at the first time-point, 144 (IQR, 101-172) at the second and 156 (IQR, 105–159) at the third.

### IgG antibody kinetic througth 9 months of following-up

**Figure 3:**
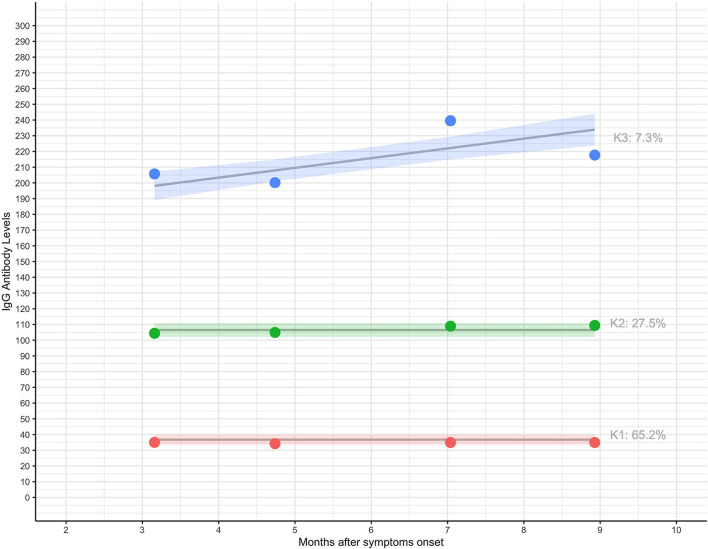
Antibody kinetics, and proportion of individuals in the groups. Shapes represent observed membership and lines represent predicted group memberships.

The IgG antibody kinetics were best represented using a 3-group model with two linear and one quadratic kinetics. Kinetics are plotted in Figure 3. Participants had three distinct IgG antibody kinetics between zero to 9 months after symptoms onset. Kinetic 1 (K1) is characterized by a constant mild antibody kinetic (group size: 65.2%); kinetic 2 (K2), composed by constant moderate antibody kinetic level (group size: 27.5%) and kinetic 3 (K3) higher antibody kinetic level (group size: 7.3%).

Participant characteristics according to antibody kinetic levels, are presented in Table 5. Looking at the characteristics of the participants, women are predominant in the three considered kinetics. Participants aged 36–55 years are predominant in the three kinetics levels. However, it is visible that older participants, aged 56 or over, are mostly found in the groups where the levels of IgG antibodies was higher (constant moderate or higher). Most overweight or obese participants are found in the groups with the highest IgG antibodies levels, contrary to those with underweight or normal weight, that are more frequent in the constant mild kinetics. Although the number of participants with two or more comorbidities is reduced, there ia a higher percentage of these participants in the kinetics with constant moderate or high IgG antibodies levels. Symptomatic disease is the most common in all groups, with the highest percentage in the group with a constant moderate levels, and the highest percentage of asymptomatic patients in the group with a constant mild levels. The number of participants with severe disease is small, with the highest percentage in the group with high levels of antibodies and the smallest in the group with constant mild levels; mild to moderate disease is the most frequent in the three groups, with the highest percentage in the group with constant mild levels of antibodies, and, this is also the group where the highest percentage of participants with severe disease is found.

**Table 5:** Baseline characteristics according to antibody concentration trajectory.

	**Constant mild antibody concentration *n =* 311**	**Constant moderate antibody concentration *n =* 128**	**Higher antibody concentration *n =* 35**
**Sex**			
Women	244 (78.5%)	100 (78.1%)	21 (60.0%)
Men	67 (21.5%)	28 (21.9%)	14 (40.0%)
**Age group (y)**			
18–35	79 (25.4%)	18 (14.1%)	5 (14.3%)
36–55	163 (52.4%)	55 (43.0%)	13 (37.1%)
56–96	69 (22.2%)	55 (43.0%)	17 (48.6%)
**BMI categories**			
Underweight/Normal (BMI < 24.99 kg/m2)	161 (52.1%)	55 (43.3%)	14 (40.0%)
Overweight/Obese (BMI ≥25 kg/m^2^)	148 (47.9%)	72 (56.7%)	21 (60.0%)
**Multimorbidity**			
0–1	251 (91.3%)	97 (84.4%)	29 (93.8%)
≥2	24 (8.7%)	18 (15.7%)	3 (9.4%)
**Type of disease**			
Asymptomatic	60 (19.29%)	19 (14.84%)	6 (17.1%)
Symptomatic	251 (80.7%)	109 (85.2%)	29 (82.9%)
**Severity**			
Asymptomatic, mild to moderate	293 (95.2%)	112 (88.2%)	28 (82.4%)
Severe	16 (45.2%)	12 (9.5%)	4 (11.8%)
Critical	1 (0.3%)	3 (2.4%)	2 (5.9%)
Antibodies (mean ± sd)	35.0 ± 23.8	104.9 ± 38.9	205.4 ± 73.9

### Health and sociodemographic factors associated with antibody kinetics groups

Table 6 presents the multinomial logistic regression model for IgG antibody levels kinetic groups. “Constant moderate” IgG antibody level kinetic group was used as the reference. People with ≥56 years old (OR: 3.33; CI 95%: [1.64; 6.67]; *p*-value: 0.001) and symptomatic SARS-CoV-2 infection (OR: 2.08; CI 95%: [1.08; 4.00]; *p*-value: 0.031) had higher odds of a “Moderate IgG antibody level kinetic”. No significant association was found regarding the “Higher IgG antibody level kinetic”.

**Table 6:** Sociodemographic and health related factors associated with antibody concentrations.

**Trajectory**		**ß**	**Sd.Err**	**OR**	**95% CI for OR**	***p*-value**
**Constant mild vs. constant moderate**						
**Sex**						
	Women	Ref	-	-	-	-
	Men	0.28	0.29	1.33	[0.75; 2.36]	0.332
**Age group (y)**						
	18–35	Ref	-	-	-	-
	36–55	−0.27	0.33	0.77	[0.41; 1.45]	0.415
	≥56	−1.20	0.35	0.30	[0.15; 0.61]	0.001
**BMI categories**						
	Underweight/Normal weight	Ref	-	-	-	-
	Overweight/Obese	−0.17	0.25	0.84	[0.52; 1.37]	0.493
**Multimorbidity**						
	0–2	Ref	-	-	-	-
	≥2	−0.21	0.37	0.81	[0.39; 1.66]	0.561
**COVID-19 symptoms**						
	Asymptomatic	Ref				
	Symptomatic	−0.73	0.40	0.48	[0.25; 0.93]	0.031
**Severity**						
	Critical	Ref				
	Assymtomatical, Mild to moderate	1.18	1.28	3.24	[0.26; 39.78]	0.358
	Severe	0.77	1.33	2.15	[0.16; 29.41]	0.565
**High vs. constant moderate**						
**Sex**						
	Women	Ref	-	-	-	-
	Men	0.87	0.46	2.39	[0.96; 5.91]	0.060
**Age group (y)**						
	18–35	Ref	-	-	-	-
	36–55	0.01	0.66	1.01	[0.28; 3.70]	0.983
	≥56	0.34	0.66	1.40	[0.39; 5.06]	0.611
**BMI categories**						
	Underweight/Normal	Ref	-	-	-	-
	Overweight/Obese	−0.06	0.45	1.12	[0.39; 2.28]	0.895
**Multimorbidity**						
	0–1	Ref	-	-	-	-
	≥2	−1.28	0.84	0.277	[0.05; 1.43]	0.126
**COVID-19 symptoms**						
	Asymptomatic	Ref				
	Symptomatic	0.3	0.59	0.65	[0.20; 2.06]	0.462
**Severity**						
	Critical	Ref				
	Asymptomatic, mild to moderate	−1.84	1.26	0.16	[0.01; 1.86]	0.143
	Severe	−1.45	1.34	0.11	[0.02; 3.28]	0.282

Reference: Constant moderate (*n* = 128).

Parameter estimates, standard error, odds ratio (OR) and respective 95% confidence intervals and *p*-values of the Model (*n* = 416).

## Discussion

This longitudinal study shows that anti-spike (anti-S) SARS-CoV-2 IgG antibodies remain detectable 9 months after SARS-CoV-2 acute infection (89.2%) for most participants. The persistence of IgG antibodies over time has been previously described in most studies where the follow-up period was 6–8 months (14–16). A few recent studies have shown that humoral immunity persists for more than 12 months after the onset of symptoms, despite a decline in antibody concentrations (17–19).

Our results suggest that IgG antibody levels in older participants (≥56 years) with severe disease are higher. Similar dynamics have also been found in other studies (16, 19–21).

Moreover, our results show that patients may have three different kinetics for the levels of IgG antibodies: “Constant lower,” “Constant moderate” and “Higher.” A small number of participants (7.3%) belong to the kinetic with the highest antibody level in the 9 months period. These participants are the same ones that have the highest antibody level at baseline on average (205.4 ± 73.9). As far as we know, there are no other works that show kinetics for the anti-SARS-CoV-2 IgG levels.

Regarding factors associated with different kinetics, in our study, age ≥56 years and symptomatic disease are associated with moderate levels IgG antibodies. If accept a good correlation between the serologic assay (anti-S) and neutralizing antibodies these asocistions point to a greater susceptibility to reinfection in younger participants and in asymptomatic patients. None of the factors tested were associated with the kinetics with the highest IgG antibody levels.

Age was previously associated with higher IgG antibody levels (18, 21, 22). Yang et al. showed a moderate but positive correlation with age in adults and the SARS-CoV-2 IgG antibody levels (21). According to these authors, this association with age was expected, given that individuals expand their catalog of memory B and T cells through accumulated immunological memory. Furthermore, the SARS-CoV-2 IgG antibody response would have been expected to occur at a more advanced age, when the aging immune system fails to mount a robust response to new antigenic challenges. One possibility may lie in the increase in comorbidities, such as obesity, hypertension, or diabetes, commonly associated with advanced age in Western society.

Regarding the levels of IgG antibodies in patients with asymptomatic and symptomatic disease, some studies point to the existence of evidence for significantly higher levels of SARS-CoV-2 spike (anti-S) protein antibodies in samples from individuals with symptomatic SARS-CoV-2 infection compared to samples from individuals who had asymptomatic infections, as well as a more sustained antibody titer that was still detectable at 7 months after SARS-CoV-2 acute infection (25). Individuals with asymptomatic infections were more likely to revert to being seronegative (25). Our results are in agreement with those presented by others (25–28). However, we still do not have the consensus on whether the symptomatic disease is associated with a more sustained concentration of neutralizing antibodies (25).

Regarding the secondary objective, an association was found between having severe disease and being male, overweight or obese, and having multimorbidity. These factors have also been described by others (29–32).

In our study, we focus on IgG antibodies because this antibody is often the most abundant in the serum and plasma, and have higher specificities when compared with assays that detect IgM; and IgG plays a more prominent role after first 2–3 weeks following acute infection, and establishing long-term immune memory, that can persist for several months or years. As our objective is to see the dynamics of antibodies over time, IgG antibodies are the most appropriate due to the characteristics presented (12, 13).

This study has some limitations. First, we are facing a convenience sample, which can introduce a bias; for example, the male/female ratio is not balanced. Second, we have few participants with severe disease, which may influence our results. On the other hand, there are also some strengths like the size of the sample, and the good characterization of the participants in the baseline.

## Conclusion

In conclusion, our results demonstrate a lasting anti-spike (anti-S) IgG antibody response at least 9 months after diagnosis in most participants with COVID-19.

Our results suggest that younger participants with asymptomatic disease have lower IgG antibody levels and are therefore probably more susceptible to reinfection.

This information contributes to expanding knowledge of SARS-CoV-2 IgG serological response and has direct implications in the adopting of preventive strategies and public health policies.

## Data availability statement

The raw data supporting the conclusions of this article will be made available by the authors, without undue reservation.

## Ethics statement

The study was carried out following the Declaration of Helsinki principles. Prior to the study beginning, an approval from the National Ethical and Deontological Committee of the Portuguese Medical Association (Conselho Nacional de Ética e Deontologia da Ordem dos Médicos) was received. All participants who agreed to take part in the study gave their written informed consent prior to their participation.

## Author contributions

Conceptualization: PQ, GS, MG, FA, and ÁC. Methodology: PQ, AR, AH, and ÁC. Validation: PQ, AR, AH, HC, and ÁC. Formal analysis: AH and AR. Investigation: PQ, JR, GS, MG, FA, and ÁC. Data curation: AH and AR. Writing—original draft preparation: AH, NM, and AR. Project administration and funding acquisition: ÁC. All authors contributed to manuscript revision, read, and approved the submitted version.
