# Supplementary material for: Persistence of IgG COVID-19 antibodies: A longitudinal analysis
Source: Front Public Health. 2023 Jan 10;10:1069898. doi: 10.3389/fpubh.2022.1069898 (PMC9872107; doi:10.3389/fpubh.2022.1069898)
Supplement: Supplementary file 1 [file Table_1.DOCX]

**Table S1**- Parameter estimates, standard error, odds ratio (OR) and respective 95% confidence intervals and p-values of the Model (Severe diseases vs moderate or mild disease) (n=513)

| **Severity** |  | **ß** | **Std.Err** | **OR** | **95% CI for**  **OR** | ***p-value*** |
| --- | --- | --- | --- | --- | --- | --- |
| **Sex** |  |  |  |  |  |  |
|  | Female | Ref | - | - | - | - |
|  | Male | 1.22 | 0.33 | 3.38 | [1.76; 6.51] | <0.001 |
| **Age group (y)** |  |  |  |  |  |  |
|  | 18-35 | Ref | - | - | - | - |
|  | 36-55 | -0.103 | 0.48 | 0.90 | [0.35; 2.31] | 0.853 |
|  | ≥56 | 0.279 | 0.50 | 1.32 | [0.50; 3.51] | 0.603 |
| **BMI categories** |  |  |  |  |  |  |
|  | Underweight/ Normal | Ref | - | - | - | - |
|  | Overweight/ Obese | 0.76 | 0.38 | 2.13 | [1.01; 4.51] | 0.049 |
| **Multimorbidity** |  |  |  |  |  |  |
|  | 0-1 | Ref | - | - | - | - |
|  | ≥2 | 1.02 | 0.42 | 2.78 | [1.23; 6.28] | 0.014 |

BMI, body mass index; Ref, reference.

**Table S2**. Bayesian Information Criterion (BIC) values and estimated group sizes (%) for the IgG trajectories model.

| **Number**  **of groups** | **Polynomial order^a^** | **BIC** | **Estimated group sizes** | | | | |
| --- | --- | --- | --- | --- | --- | --- | --- |
| 1 | 2 | -8762.39 | 100 |  |  |  |  |
| 1 | 1 | -8759.45 | 100 |  |  |  |  |
| 2 | 11 | -8307.01 | 82.77 | 17.23 |  |  |  |
| 3 | 111 | -8060.90 | 65.38 | 27.38 | 7.24 |  |  |
| 3 (final) | 001 | -8055.04 | 65.23 | 27.51 | 7.26 |  |  |

**Table S3**. IgG trajectory model diagnostic criteria.

|  | **n** | **Estimated group probabilities** | **Proportion classified^a^** | **APP^b^** | **OCC^c^** |
| --- | --- | --- | --- | --- | --- |
| **IgG Antibody Group** |  |  |  |  |  |
| Constant Mild | 311 | 0.652 | 0.656 | 0.972 | 18.20 |
| Constant Moderate | 128 | 0.275 | 0.270 | 0.944 | 45.45 |
| Higher | 35 | 0.073 | 0.074 | 0.975 | 486.67 |

**^a^** Estimated group probabilities should be close to the proportion of individuals classified in the group (proportion based on the assignments for the maximum posterior probability). **^b^** Average Posterior Probabilities (should be at least 0.7). **^c^** Odds of Correct Classification (should be ³5.0).
